# An evidence informed framework for artificial intelligence in rare breast cancers using small cohort validation synthetic data practices and clinical governance

**DOI:** 10.1007/s12672-026-05573-1

**Published:** 2026-07-19

**Authors:** Bandar Saad Alshreef

**Affiliations:** Department of Clinical Laboratory Sciences, College of Applied Medical Sciences, Shaqra University, Shaqra, Saudi Arabia

**Keywords:** Artificial intelligence, Rare breast cancers, Synthetic data, Small-cohort validation, Clinical governance, Precision oncology, Machine learning, Breast cancer, External validation, Lifecycle monitoring

## Abstract

Rare breast cancers represent a clinically important but underrepresented group of malignancies. In this Perspective, rare breast cancers are considered within the broader rare cancer definition of an annual incidence below 6 cases per 100,000 persons, while also recognizing breast-specific rarity based on uncommon histology, molecular hallmarks, clinical presentation or sex-specific occurrence. These conditions are characterized by limited case numbers, biological heterogeneity, reduced clinical trial inclusion and fragmented evidence. These constraints challenge artificial intelligence (AI) development because many systems depend on large, balanced and externally validated datasets. AI may support diagnosis, histopathology, molecular interpretation, prognostic stratification and precision oncology decision support, but its use in rare breast cancers requires evidence standards adapted to small cohorts. This Perspective proposes an evidence-informed clinical governance framework organized around five domains: intended clinical use, small-cohort validation, synthetic data governance, human oversight and lifecycle monitoring. Its distinctive contribution is to translate general AI reporting and governance principles into rare breast cancer-specific safeguards, including objective data-quality checks, leakage prevention, uncertainty-aware validation, synthetic data plausibility scoring, pan-rare model reporting, patient involvement and post-deployment surveillance. Synthetic data may support development and simulation, but should not replace validation on real clinical cases. By linking small-cohort methodology with clinical oversight, regulatory alignment and lifecycle monitoring, the framework offers a practical roadmap for safe AI-enabled rare breast cancer precision oncology.

## Plain Language summary

Rare breast cancers are difficult to study because they occur infrequently and are often missing from large clinical datasets. This creates problems for artificial intelligence because AI usually needs large and diverse data to work reliably. Synthetic data may help with training and simulation, but it can also create misleading results if it is not carefully reviewed and separated from validation data. This article proposes a stepwise governance approach for using AI safely in rare breast cancers, with emphasis on clear clinical purpose, careful validation in small cohorts, responsible synthetic data use, expert human oversight and continuous monitoring after implementation.

## Key points

- Rare breast cancers challenge conventional AI development because small sample sizes, class imbalance, heterogeneous biology and limited external validation increase the risk of overfitting and misleading performance claims.
- This Perspective defines rare breast cancers using the broader rare cancer threshold of < 6 cases per 100,000 persons per year, together with breast-specific rarity based on uncommon histology, molecular phenotype, clinical presentation or sex-specific occurrence.
- Synthetic data may support AI development in rare breast cancer research, but it requires strict governance, including provenance transparency, clinical plausibility review, leakage prevention, privacy assessment and validation on real clinical data.
- The proposed five-domain framework links intended clinical use, small-cohort validation, synthetic data governance, human oversight and lifecycle monitoring to support safe and accountable AI-enabled precision oncology.
- Independent external cohorts are often infeasible in ultra-rare settings; credible alternatives include temporal validation, leave-one-institution-out validation, prospective registry monitoring and cautious claim limitation.

## Introduction

Rare breast cancers represent a clinically important but historically underrepresented group of malignancies. For the purposes of this Perspective, rarity is defined using the widely adopted rare cancer threshold of an annual incidence below 6 cases per 100,000 persons, while recognizing that breast-specific rarity may also arise from uncommon histology, molecular phenotype, clinical presentation or sex-specific occurrence [26]. Although breast cancer is one of the most extensively studied cancers worldwide, uncommon histological and clinical presentations such as metaplastic, neuroendocrine, inflammatory, medullary, secretory and male breast cancers remain difficult to study at scale. The target Collection, Demystifying Rare Breast Cancers, explicitly emphasizes this gap and highlights artificial intelligence (AI), machine learning, molecular profiling, novel therapeutic strategies and small-cohort research designs as areas of interest [1].

This classification is operational rather than absolute. Some entities may be rare because of histological subtype, some because they occur in a usually underrepresented population, and others because molecular stratification divides a common cancer into biologically small subgroups. This matters for AI because a model trained on common invasive ductal carcinoma cannot automatically be assumed to generalize to metaplastic, inflammatory, neuroendocrine or male breast cancers. Rare breast cancers therefore require explicit subtype definitions, harmonized labels and evidence claims limited to the population actually studied.

Rarity affects every stage of the evidence pathway. At diagnosis, clinicians may encounter atypical radiological or pathological appearances that are less familiar than common invasive ductal and lobular carcinomas. At the molecular level, uncommon breast cancer subtypes may show distinctive genomic, receptor, immune or tumor microenvironment features that influence prognosis and therapeutic sensitivity. At the research level, limited case numbers restrict large prospective trials, independent external validation cohorts and high-confidence subgroup-specific recommendations [2, 3].

AI offers an opportunity to address some of these constraints. In broader breast cancer care, AI has been applied to mammography, magnetic resonance imaging, digital pathology, molecular subtype prediction, biomarker discovery, prognosis and workflow support [6–8]. In rare breast cancers, AI could support earlier recognition of atypical imaging patterns, assist histopathological review, integrate molecular and clinical information and strengthen multidisciplinary tumor board decision-making. However, the same rarity that makes AI attractive also increases the risk of overfitting, unstable feature selection, biased performance estimates and premature clinical claims.

Rare breast cancers are a uniquely urgent use case for AI because the clinical problem is not simply data scarcity. These cancers often combine diagnostic unfamiliarity, limited specialist exposure, inconsistent subtype definitions, molecular heterogeneity, low trial representation and high uncertainty at the point of care. AI may be useful precisely when it helps retrieve similar cases, integrate multimodal evidence, flag atypical patterns or support expert referral. However, these same features make ungoverned AI particularly risky: apparent performance may reflect local artifacts, pooled-subtype shortcuts or leakage rather than transferable biological signal.

This Perspective argues that AI for rare breast cancers should not be treated only as a technical modeling problem. It is also a clinical governance challenge. Existing AI reporting and governance standards are valuable, but they are not tailored to ultra-rare oncology cohorts, pan-rare pooled models or synthetic data use in rare breast cancer settings. The proposed evidence-informed framework links intended clinical use, small-cohort validation, synthetic data governance, human oversight and lifecycle monitoring. The remainder of this Perspective is structured around these five domains, followed by an implementation roadmap and a reporting checklist for rare breast cancer AI studies.

### Rare breast cancers as a precision oncology challenge

Rare breast cancers are not a single biological entity. They represent a heterogeneous group of uncommon histological, molecular and clinical subtypes that fall outside the dominant evidence base built around common breast cancer presentations. Within an incidence-based rare cancer framework, rarity should be interpreted alongside breast-specific diagnostic categories, molecular hallmarks and clinical context because the evidence problem differs across metaplastic, inflammatory, neuroendocrine, secretory and male breast cancers [2, 3, 26]. Recent reviews emphasize that special histologic subtypes have distinctive phenotypes and molecular profiles with diagnostic and therapeutic implications, and that uncommon clinical presentations such as male and inflammatory breast cancer create specific challenges [2, 3]. Representative subtypes, potential AI contributions and governance caveats are summarized in Table 1.

**Table 1:** Rare breast cancer subtypes and potential AI applications

Rare subtype / presentation	Core challenge	Potential AI contribution	Governance caveat
Metaplastic breast cancer	Aggressive biology, limited trials, heterogeneous histology	Imaging-pathology integration, prognostic modeling, case similarity search	Require subtype-confirmed labels and external testing where feasible
Inflammatory breast cancer	Rapid progression and atypical presentation	Triage, imaging pattern recognition, workflow prioritization	Avoid replacing urgent clinical assessment
Neuroendocrine breast carcinoma	Diagnostic ambiguity and limited evidence	Pathology decision support and molecular correlation	Require expert pathology review and differential diagnosis safeguards
Secretory carcinoma	Very low case numbers and molecular distinctiveness	Registry-based learning and molecular-pathology support	Do not overstate performance from tiny cohorts
Medullary / special histologic subtypes	Variable definitions across studies	Subtype classification support and feature discovery	Use harmonized diagnostic criteria
Male breast cancer	Low awareness, delayed diagnosis, limited trial inclusion	Risk stratification, imaging support, registry analytics	Report sex-specific performance rather than assuming transferability
Pregnancy-associated or other rare presentations	Clinical complexity and data scarcity	Decision-support summaries and expert referral prompts	Require conservative deployment and expert oversight
Pan-rare pooled models	Multiple rare subtypes may be combined despite distinct biology and different error consequences	Shared representation learning, case similarity search and hypothesis generation across ultra-rare groups	Report subtype-specific confusion matrices, calibration and claim limitations rather than relying on pooled metrics

Male breast cancer illustrates how rarity complicates care. The World Health Organization notes that approximately 0.5-1% of breast cancers occur in men, while public health sources similarly describe male breast cancer as about 1 in 100 diagnosed breast cancers in the United States [4, 5]. This rarity contributes to reduced awareness, delayed recognition, limited trial inclusion and uncertainty over how far evidence from female breast cancer populations can be generalized.

For precision oncology, the challenge is intensified by multimodal decision-making. Rare breast cancer management may require integration of imaging, histopathology, immunohistochemistry, genomic testing, treatment history and outcomes. For example, a patient with inflammatory breast cancer may require MRI interpretation, core biopsy confirmation, HER2 and PD-L1 assessment, genomic testing and urgent multidisciplinary treatment planning. AI-driven multi-omics and computational pathology approaches may help connect these data streams, but they require high-quality labels, sufficient case diversity and rigorous validation [8, 9]. In rare breast cancers, incomplete data, inconsistent subtype definitions and variable molecular testing practices may weaken model credibility before modeling begins.

Therefore, rare breast cancer AI requires a development model that is different from common breast cancer AI. The central question is not only whether a model can achieve a high area under the receiver operating characteristic curve in a small retrospective dataset. The more important question is whether its outputs are clinically meaningful, externally credible, transparently reported, accountable to human expertise and monitored after implementation.

### Artificial intelligence opportunities in rare breast cancer care

AI may add value across the rare breast cancer care pathway. In imaging, AI could help identify atypical mammographic, ultrasound or MRI patterns, prioritize suspicious cases for expert review and support radiologists in settings where rare phenotypes are infrequently encountered. The international evaluation of an AI system for breast cancer screening demonstrated the broader potential of AI in mammography, but rare subtype performance should not be assumed unless specifically tested [6].

Digital pathology is another promising domain. Whole-slide imaging allows AI systems to analyze tissue architecture, nuclear features, mitotic activity, stromal patterns, immune infiltration and tumor heterogeneity. Recent work in breast computational pathology highlights opportunities for predicting clinically relevant molecular features such as hormone receptor status, HER2, Ki-67, BRCA-related status and somatic mutations from whole-slide images, while also emphasizing workflow integration, explainability and generalizability barriers [9].

Molecular interpretation and precision decision support are particularly relevant to rare cancers. AI may help organize complex clinical and genomic information, identify biologically similar cases, support trial matching and assist molecular tumor boards in synthesizing evidence. Recent molecular AI and AlphaFold-related research illustrates the accelerating role of machine learning in structural biology and drug discovery, while rare breast cancer molecular studies such as triple-negative breast cancer brain metastasis biology show why subtype-specific evidence remains necessary [27, 28]. Foundation models, large language models and multimodal pathology systems may accelerate diagnostic, prognostic and biomarker prediction tools, but transfer learning from common cancers cannot be considered proof of safety in rare subtypes, and generative outputs require verification before clinical use [10, 11, 29].

**Fig. 1:**
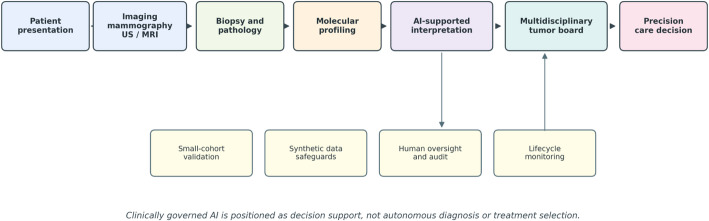
AI-enabled rare breast cancer precision oncology workflow. The workflow illustrates how AI can support rare breast cancer care across presentation, imaging, biopsy, pathology, molecular profiling, multidisciplinary tumor board review and precision care decisions. The bottom row summarizes governance safeguards that should accompany model development and deployment, including intended-use control, small-cohort validation, synthetic-data governance, human oversight and lifecycle monitoring

The most realistic near-term role for AI is not autonomous diagnosis or treatment selection. Rather, AI should function as clinically governed decision support: a system that improves recognition, prioritization, information integration and multidisciplinary interpretation while leaving final responsibility with clinicians and tumor boards. Figure 1 summarizes how AI-enabled decision support can align with the rare breast cancer precision oncology workflow, from presentation and diagnostic work-up through tumor board review, clinical decision-making and lifecycle monitoring.

### The small-cohort validation problem

The central methodological problem is that rare breast cancer datasets are small, imbalanced and heterogeneous. Standard AI workflows often assume access to large, diverse and well-labeled datasets with enough events for training, hyperparameter tuning, internal validation and independent external testing. Rare breast cancers rarely satisfy these conditions. As a result, model performance can appear stronger than it truly is.

Overfitting is a major risk. When the number of candidate variables, imaging features, pathology descriptors or molecular markers is large relative to case numbers, models may learn dataset-specific noise rather than transferable biological signal. Practical safeguards include pre-specifying clinically plausible features, restricting feature dimensionality, using penalized or Bayesian models where appropriate, comparing against simpler baselines, and applying nested validation to avoid feature-selection leakage. These safeguards are especially relevant for radiomics, deep learning, computational pathology and multi-omics, where thousands of candidate features can be extracted from limited cases.

Class imbalance can also hide clinically important failure. A dataset may include many common breast cancers but very few rare subtype cases. A model may perform well overall while performing poorly in the rare subgroup that is clinically most important. Therefore, aggregate AUC or accuracy is insufficient. Rare-subtype-specific sensitivity, specificity, precision, recall, calibration, uncertainty and error analysis should be reported whenever possible.

Metric selection should be matched to the clinical endpoint and the size of the rare subgroup. In highly imbalanced datasets, conventional AUC can be unstable and clinically misleading if minority-class event counts are very small. Therefore, AUC should be accompanied by confidence intervals, precision-recall metrics, rare-subtype sensitivity, calibration slope or intercept, Brier score, decision-curve analysis where appropriate and explicit reporting of event counts. For survival outcomes, concordance index values should be reported with uncertainty intervals and, where feasible, time-dependent AUC and calibration at clinically meaningful time points. These statistics should be interpreted as uncertainty-aware evidence rather than definitive proof of generalizability.

Leakage prevention should be explicit and auditable. Patient-level separation must be maintained across training, validation and test sets, and all preprocessing, feature selection, augmentation, imputation, normalization and hyperparameter tuning should be performed inside the training loop. When data are pooled across hospitals, site-aware splitting and leave-one-institution-out testing can reduce the risk that models learn institutional signatures rather than rare breast cancer biology.

Internal validation methods such as cross-validation, bootstrapping and nested cross-validation are useful but cannot replace external validation. TRIPOD + AI provides updated reporting guidance for clinical prediction models using regression or machine learning methods, while the 2024 CLAIM update provides dedicated guidance for medical imaging AI studies [12, 13]. The checklist later summarized in Table 5 adapts these standards to rare breast cancer-specific issues, including subtype definition, pan-rare pooling, external validation alternatives and synthetic data governance.

To reduce reliance on subjective expert opinion, biological coherence and data readiness should combine multidisciplinary review with objective quantitative checks. For tabular clinical or registry data, investigators may assess distributional similarity using standardized mean differences, Mahalanobis distance, Kullback-Leibler or Jensen-Shannon divergence, maximum mean discrepancy, population stability index and missingness patterns. For imaging and digital pathology, quality and similarity checks may include structural similarity index, peak signal-to-noise ratio, radiomics feature distribution shift, embedding-space distance, scanner or staining artifact audits and segmentation-quality indicators. These measures do not replace clinical judgment, but they make the scoring system more reproducible and help distinguish clinically plausible signal from local artifacts.

When truly independent external cohorts are unavailable, investigators should report the best feasible validation design rather than treating absence of external validation as a minor limitation. Minimum alternatives include temporal validation using later cases from the same institution, leave-one-institution-out validation within a consortium, validation across scanner or pathology workflow changes, silent prospective deployment, and prospective registry-based monitoring as rare cases accrue. These designs do not provide the same evidentiary strength as fully independent external testing, but they create a more credible pathway than repeated internal resampling alone.

A related challenge is the development of multi-class or pan-rare models that combine several uncommon subtypes into a single system. Such models may be statistically attractive because they increase the total number of rare cases, but they can obscure subtype-specific failure. A pan-rare model should therefore report confusion matrices, calibration and error analysis for each subtype whenever feasible, and should avoid presenting pooled performance as evidence of reliability for every rare cancer category.

Explainability should also be interpreted cautiously in small cohorts. Post-hoc saliency maps or feature importance outputs may be unstable when the model is underpowered or trained on highly imbalanced data. In high-stakes rare cancer settings, inherently interpretable models, pre-specified features, uncertainty estimates and expert review may be preferable to complex black-box systems when the available data cannot support reliable deep learning explanations [25]. Key risks and corresponding governance safeguards are summarized in Table 2.

**Table 2:** Risks of AI in rare breast cancer and governance safeguards

Risk	Why it matters	Example safeguard
Small sample size	Performance estimates may be unstable and confidence intervals wide	Report event counts, uncertainty intervals and evidence limitations
Class imbalance	Overall accuracy may hide rare-subtype failure	Report rare-subtype-specific sensitivity, specificity and error analysis
Overfitting	Model may learn noise, artifacts or local practice patterns	Use nested validation, bootstrapping, simpler baselines and external testing
Label inconsistency	Rare subtype misclassification can invalidate model training	Use expert-confirmed pathology and multidisciplinary adjudication
Synthetic data leakage	Synthetic cases may indirectly expose validation/test information	Separate source, training, validation and test data at patient level
Artificial realism	Generated cases may look plausible but distort disease biology	Require clinical plausibility review by domain experts
Bias and poor generalizability	Underrepresented populations may receive less reliable outputs	Assess subgroup performance and dataset diversity
Post-deployment drift	Scanner, staining, molecular panel or population changes may degrade performance	Monitor data drift, performance drift and safety events
Limited external cohorts	Independent validation may be infeasible for ultra-rare subtypes	Use temporal validation, leave-one-institution-out validation, registry-based monitoring and cautious claim limitation
Pan-rare model pooling	Pooled performance can hide subtype-specific failure	Report confusion matrices, subtype-specific metrics and calibration where feasible
Unstable explanations	Post-hoc saliency maps may be unreliable in underpowered models	Prefer interpretable models when appropriate and require expert plausibility review
Limited patient involvement	AI risk thresholds may not reflect patient priorities	Include patient perspectives in governance, communication and acceptable-risk decisions
Regulatory fragmentation	Rare cancer AI may be deployed across jurisdictions with different requirements	Align evidence, monitoring and documentation with FDA/IMDRF, WHO and EU MDR/IVDR principles

### Synthetic data and generative AI in rare breast cancer research

Synthetic data and generative AI are attractive because rare breast cancers are difficult to study at scale. Synthetic mammography, pathology-like images and structured clinical datasets may support data augmentation, class balancing, simulation and privacy-preserving collaboration. A 2025 review of synthetic data and generative AI in breast imaging concluded that these methods may address data scarcity, privacy restrictions and uneven representation, but require rigorous validation, transparency, equity auditing and shared accountability [14].

The risks are amplified in rare breast cancers. The source data used to generate synthetic cases may already be incomplete, biased or poorly representative of true disease variability. A generative model may reproduce scanner signatures, staining artifacts, institution-specific labels or demographic imbalance rather than clinically meaningful rare cancer biology. Synthetic cases may appear realistic while failing to preserve tumor margins, receptor patterns, stromal reaction, immune contexture or treatment response.

Therefore, synthetic data should be viewed as a supplementary development tool, not as a substitute for real rare cancer evidence. It may support model training, class balancing, simulation and stress testing, but claims of clinical performance should remain grounded in real clinical validation. Models evaluated exclusively on synthetic test sets should not be described as clinically validated, regardless of apparent performance.

A strict separation should be maintained between source data used to train generative models, synthetic data used for augmentation, internal validation data, external test data and prospective clinical evaluation data. Synthetic data leakage is particularly dangerous in rare cancers because a small number of real cases may be repeatedly reused across development and evaluation. Expert radiology, pathology, molecular and oncology review should assess not only whether synthetic data look realistic, but whether they are biologically coherent. Synthetic data review should therefore combine expert plausibility scoring with quantitative fidelity, diversity, privacy and utility checks 15 . For tabular synthetic records, distributional metrics, nearest-neighbor distance, membership-inference or memorization testing and subgroup balance should be reported. For synthetic imaging or pathology, structural similarity, feature-distribution shift, radiomics stability, embedding-space similarity and artifact audits should be considered. Approval for use should depend not only on visual realism, but also on whether any performance gain persists when tested on independent real clinical cases.

To operationalize clinical plausibility review, this framework recommends a structured discipline-specific scoring process for synthetic rare breast cancer data. Reviewers should not only judge whether an image or record appears realistic; they should assess whether the generated case is biologically coherent for the stated rare subtype. For example, imaging appearance, histological features, receptor status, molecular markers, stage, treatment pathway and outcome pattern should be compatible. A synthetic case that looks visually plausible but contradicts expected subtype biology should not be used to support clinical performance claims.

A pragmatic scoring rubric should include technical fidelity, subtype-specific biological coherence, artifact and bias risk, privacy and memorization risk, and downstream utility on real validation data. Each domain can be rated by relevant experts such as breast radiologists, pathologists, molecular pathologists, oncologists, data scientists and clinical AI governance teams. Objective metrics should be documented alongside expert scores so that clinical plausibility review becomes reproducible rather than symbolic. Table 3 summarizes the proposed scoring rubric for synthetic rare breast cancer data.

**Table 3:** Pragmatic clinical plausibility scoring rubric for synthetic rare breast cancer data

Domain	Scoring focus	Suggested reviewer	Minimum acceptable evidence
Technical fidelity	Image, slide or record quality; realism; absence of obvious artifacts	Radiologist, pathologist or data scientist	Synthetic cases are technically usable and free of obvious generation artifacts
Subtype-specific biological coherence	Compatibility between rare subtype, imaging pattern, histology, receptor profile, molecular features and clinical course	Breast pathologist, molecular pathologist and oncologist	Generated cases are clinically plausible for the declared rare subtype
Bias and artifact risk	Scanner signatures, staining artifacts, demographic imbalance or institution-specific labels	Clinical AI governance team	No unacceptable artifact-driven or subgroup-biased patterns are identified
Privacy and memorization	Near-duplicate generation or patient-level memorization	Data protection and technical review team	Nearest-neighbor and memorization checks do not suggest re-identification risk
Downstream utility	Whether synthetic data improve robustness when tested on real validation data	Model development and clinical review team	Any performance gain is confirmed on real clinical cases rather than synthetic test data
Approval decision	Permitted use for training, simulation or stress testing	Multidisciplinary review board	Use is approved only for predefined purposes and does not replace real-world validation

Suggested scale: 1 = unacceptable, 2 = major concerns, 3 = acceptable with revision, 4 = acceptable, and 5 = strong evidence

### Proposed clinical governance framework

**Fig. 2:**
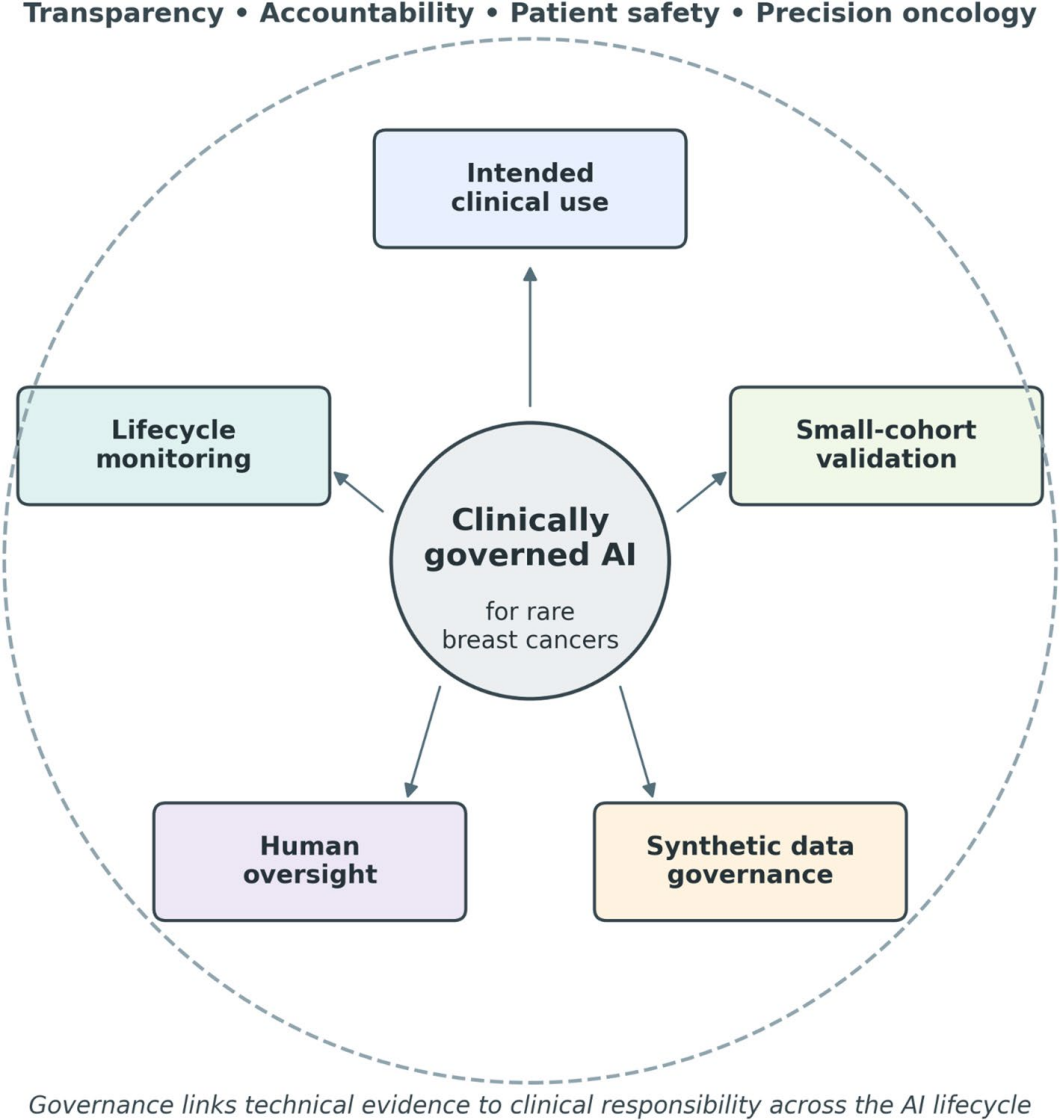
Five-domain clinical governance framework for AI in rare breast cancers. The framework positions AI as a clinically governed decision-support system organized around intended clinical use, small-cohort validation, synthetic data governance, human oversight and lifecycle monitoring. The outer ring emphasizes transparency, accountability, patient safety and precision oncology

The proposed framework includes five domains: intended clinical use, small-cohort validation, synthetic data governance, human oversight and lifecycle monitoring. As shown in Fig. 2, the framework centers on clinically governed AI and links each domain to the shared goals of transparency, accountability, patient safety and precision oncology. Compared with general AI guidance, the framework adds rare breast cancer-specific requirements for ultra-rare validation alternatives, synthetic data plausibility scoring, pan-rare model reporting and patient-centered governance.

First, intended clinical use must be explicit. The manuscript or deployment dossier should define the rare subtype, clinical setting, input data, output, target user, supported decision and limitations. A model designed to flag atypical imaging appearances should not be described as a treatment-planning system unless separately validated for that purpose. Practice point: deployment dossiers should state the maximum safe scope of use and list contraindications, including untested subtypes, unscreened populations or unsupported workflows.

Second, small-cohort validation should be uncertainty-aware. Performance should include confidence intervals, calibration, threshold-specific metrics, subtype-specific error analysis and external validation where feasible. A high AUC from a small single-center dataset should be interpreted cautiously if uncertainty intervals are wide, class balance is poor, calibration is absent or external testing is unavailable. Practice point: validation claims should be explicitly labeled as exploratory, pilot, externally validated or clinically monitored according to the evidence available.

Third, synthetic data governance should document provenance, generation method, clinical plausibility review, data separation, utility on real validation data, privacy protection and bias assessment. Synthetic data may support development, but real clinical data should remain the basis for claims about diagnostic, prognostic or treatment-planning performance. Practice point: a synthetic-data appendix should identify source data, permitted use, plausibility scores, privacy checks and whether performance gains persisted on real validation data.

Fourth, human oversight must be active. AI outputs should be reviewed by the relevant specialist: breast radiologists for imaging outputs, pathologists for histological outputs, molecular pathologists or genomic scientists for molecular interpretation and oncologists or tumor boards for treatment relevance. Governance should define when clinicians are expected to override AI, including discordance with multimodal evidence, low-confidence outputs or use outside the validated population. Overrides and disagreements should be logged for safety learning, recalibration and audit.

Patient and public involvement should also be incorporated into governance. Patients with rare breast cancers often experience diagnostic delay, uncertainty, limited evidence and psychological burden. Their perspectives can help define acceptable risk thresholds, communication preferences, consent expectations, tolerance for AI uncertainty and priorities for equitable access to expert review. Patient involvement is particularly important when AI outputs may influence referral, trial matching or treatment discussion.

Fifth, lifecycle monitoring should be planned before deployment. Model behavior may change because of scanner replacement, staining protocol changes, new molecular testing panels, changing case mix, software updates or new treatments. Monitoring should include data drift, performance drift, subgroup safety, workflow impact, user behavior, false negatives, false positives and model update governance. FUTURE-AI emphasizes trustworthy healthcare AI across development, validation, deployment and monitoring, while FDA and IMDRF materials on AI/ML-enabled medical devices emphasize lifecycle oversight and good machine learning practice [16–18].

The framework is intended to be internationally adaptable. In addition to FDA and IMDRF lifecycle principles, it is compatible with WHO ethics and governance guidance for AI in health and with European regulatory concepts for medical devices and in vitro diagnostics, including risk classification, clinical evidence, performance evaluation, post-market surveillance and transparency obligations under the EU MDR and IVDR [22–24].

### Implementation roadmap

Hospitals, academic cancer centers and rare cancer networks should follow a staged implementation roadmap. The first step is to define the use case. Rare breast cancer is not a single target condition; an AI tool should specify whether it addresses metaplastic, inflammatory, neuroendocrine, secretory, medullary, male or another rare subtype, and whether it supports imaging, pathology, molecular interpretation, prognosis or treatment planning.

The second step is to build a clinically curated data foundation. Each case should be reviewed for diagnostic accuracy, subtype definition, imaging quality, pathology quality, biomarker profile, molecular testing and relevant outcomes. Data curation should be multidisciplinary because rare subtype misclassification can invalidate downstream modeling.

The third step is governance review before model development. Ethics, privacy, data access, cybersecurity, validation strategy, synthetic data use, human oversight and escalation pathways should be reviewed before modeling begins. The fourth step is model development with small-cohort safeguards, including leakage prevention, nested validation, stability analysis, careful feature reduction, appropriate explainability and interpretable outputs where possible.

The fifth step is validation with uncertainty and clinical relevance, including temporal, external, leave-one-institution-out or prospective registry-based approaches where feasible. The sixth step is a supervised clinical pilot, ideally as silent deployment, second-read support or tumor board support before clinical influence; pilot outcomes should include decision confidence, diagnostic timing, additional investigations, workflow burden and clinician override patterns. The seventh step is governed deployment and routine use with standard operating procedures, user training, override mechanisms and documentation. The eighth step is lifecycle monitoring for drift, safety events, subgroup performance and model updates. The staged implementation roadmap is summarized in Table 4.

**Table 4:** Operational roadmap for implementation in hospitals, academic cancer centers and rare cancer networks

Phase	Main activity	Documentation artifact
1. Use-case definition	Define rare subtype, workflow, target user, input data and supported decision	Intended-use statement
2. Data foundation	Curate imaging, pathology, molecular, clinical and outcome data	Quality-controlled dataset
3. Governance review	Review ethics, privacy, cybersecurity, validation plan and synthetic data protocol	Governance approval
4. Model development	Train model using leakage prevention, stability analysis and small-cohort safeguards	Versioned AI prototype
5. Validation	Evaluate uncertainty, calibration, subgroup performance and external testing where feasible	Validation dossier
6. Clinical pilot	Test in silent deployment, second-read or tumor board support mode	Pilot evaluation report
7. Governed deployment and routine use	Implement SOPs, user training, documentation, override mechanisms and routine-use governance	Supervised decision-support workflow
8. Lifecycle monitoring	Track drift, safety events, subgroup performance and model updates	Monitoring dashboard and update policy

### Use-case application to metaplastic breast cancer

A practical use case may involve a tertiary oncology center evaluating an AI decision-support tool for metaplastic breast cancer, an uncommon subtype characterized by heterogeneous histology, aggressive behavior and limited trial evidence. The institution first defines a narrow intended use: assisting the multidisciplinary tumor board in identifying patients who may benefit from intensified imaging review, molecular profiling or referral to a specialist rare breast cancer service. The tool is not approved for autonomous diagnosis or treatment selection.

The local dataset is curated by breast radiologists, pathologists, molecular pathologists and oncologists. Each case is checked for subtype-confirmed pathology, imaging quality, receptor status, molecular testing, stage, treatment course and outcome availability. Before modeling, the team documents class imbalance, missingness, rare-subtype event counts and potential site-specific artifacts. Any synthetic data are used only for training augmentation or stress testing and are kept separate from real validation data.

During validation, the team reports rare-subtype sensitivity, precision-recall metrics, calibration, confidence intervals, false-negative review and decision-curve analysis rather than relying on a single AUC. If external validation is not feasible, temporal validation and prospective registry monitoring are used and the claim is labeled as exploratory decision support. In clinical pilot use, outputs are presented to the tumor board with uncertainty intervals, known failure modes and an override mechanism. Disagreements between clinicians and the model are logged for audit, recalibration and safety review.

### Reporting checklist for AI studies in rare breast cancers

Transparent reporting is a safety requirement. The strength of the claim must match the strength of the evidence. A retrospective single-center model should be described as exploratory. A model without external validation should not be described as clinically ready. A model trained with synthetic data should not claim patient-facing performance unless tested on independent real clinical cases.

The proposed checklist adapts principles from TRIPOD + AI, CLAIM 2024, FUTURE-AI, CONSORT-AI and SPIRIT-AI to the rare breast cancer context [12, 13, 16, 19 ,20].It also incorporates recommendations on transparent dataset documentation and the assessment and mitigation of algorithmic bias [21]. Its added value is the focus on rare subtype definition, small-cohort uncertainty, external validation alternatives for ultra-rare conditions, synthetic data plausibility scoring, pan-rare pooled model reporting, patient involvement, human oversight and lifecycle accountability. It can be used as a supplement to existing reporting checklists, a protocol template, or an institutional review aid for rare breast cancer AI projects. Table 5 presents the minimum reporting checklist for rare breast cancer AI studies.

Authors should report the rare subtype definition, inclusion and exclusion criteria, dataset sources, sample size, event counts, class balance, reference standard, data modality, data quality, preprocessing, model development, leakage prevention, internal and external validation, performance metrics, uncertainty, calibration, subgroup analysis, error analysis, synthetic data disclosure, privacy and bias assessment, explainability, human oversight, workflow integration, lifecycle monitoring and limitations. Detailed confusion matrices, calibration plots, synthetic-data privacy checks and subgroup analyses can be placed in online supplements when journal space is limited.

**Table 5:** Minimum reporting checklist for AI studies in rare breast cancers

Domain	Minimum reporting item	Rationale
Rare subtype definition	Define subtype, diagnostic criteria and inclusion/exclusion criteria	Prevents vague grouping of biologically different cancers
Clinical use case	State whether AI supports diagnosis, triage, pathology review, molecular interpretation, prognosis, treatment planning or trial matching	Ensures validation matches clinical risk
Data source and sample size	Report institution/registry source, total cases, rare subtype cases, comparators and event counts	Allows assessment of credibility and generalizability
Reference standard	Describe how diagnosis or outcomes were confirmed	Rare subtype misclassification can invalidate the model
Data modality and quality	Specify imaging, pathology, molecular, clinical or multimodal inputs, plus missingness and quality variation	Clarifies what the model learns from
Preprocessing and model development	Describe preprocessing, annotation, feature extraction, feature selection, algorithm, tuning and software environment	Supports reproducibility
Leakage prevention	Confirm patient-level separation of training, validation and test sets	Prevents inflated performance
Validation	Report internal validation, external validation where feasible, calibration and clinically relevant thresholds	Moves beyond single-point AUC claims
Uncertainty and subgroup analysis	Report confidence intervals, rare-subtype metrics and relevant demographic or institutional subgroup performance	Supports patient safety and equity
Error analysis	Analyze false positives, false negatives and uncertain predictions	Identifies clinically serious failures
Synthetic data disclosure	Report whether synthetic data were used, for what purpose and how they were evaluated	Prevents hidden augmentation or synthetic test-set claims
Synthetic data governance	Document provenance, generation method, data separation, clinical plausibility review, utility on real data and privacy/bias assessment	Reduces leakage, artificial realism and privacy risks
Explainability and uncertainty display	Describe interpretability outputs, saliency maps, feature importance, uncertainty scores or model cards	Supports clinician interpretation
Human oversight	Define who reviews AI output and who remains clinically accountable	Prevents unsafe automation
Workflow integration	Explain how AI enters radiology, pathology, molecular board or oncology workflow	Determines real-world usability
Lifecycle monitoring	Describe drift monitoring, incident reporting, update policy and thresholds for review	Maintains safety after deployment
Limitations	State limitations from small sample size, retrospective design, missing data, lack of external validation or synthetic data use	Prevents overclaiming
External validation alternatives	If independent external validation is not feasible, report temporal, leave-one-site-out, consortium-based or prospective registry-based validation.	Clarifies the evidentiary strength of small-cohort studies.
Pan-rare model reporting	Disclose whether multiple rare subtypes were pooled and report subtype-specific metrics where possible.	Prevents pooled performance from masking subtype failure.
Explainability reliability	Report whether saliency, attention or feature-importance outputs were assessed for stability and clinical plausibility.	Avoids overinterpreting unstable post-hoc explanations.
Patient and public involvement	Report whether patient perspectives informed risk thresholds, communication, consent or workflow design.	Improves acceptability and transparency.
Regulatory alignment	State whether the intended use may fall under medical-device or in-vitro diagnostic regulatory pathways and how monitoring will be documented.	Supports international translation and lifecycle accountability.

For pan-rare models, authors should report whether multiple rare subtypes were pooled for training, how each subtype contributed to the dataset, and whether subtype-specific performance could be estimated. If subtype-specific calibration or sensitivity cannot be estimated because of extremely small numbers, the manuscript should explicitly state that limitation and avoid presenting pooled metrics as evidence for all subtypes.

For explainability, authors should distinguish between interpretability as a model property and post-hoc explanation as an auxiliary output. In small-cohort rare cancer AI, saliency maps, attention heatmaps and feature importance plots should be treated as hypothesis-generating unless their stability and clinical plausibility are assessed by experts.

### Future directions

The future of AI for rare breast cancers will depend on collaborative infrastructure rather than model complexity alone. International rare breast cancer registries should link clinical, imaging, pathology, molecular, treatment and outcome data using consistent subtype definitions and quality metadata. Such registries would support larger development cohorts, more credible external validation and better subgroup performance evaluation.

Federated learning may be useful because it allows models to learn across institutions without centralizing patient-level data. For rare breast cancers, site-level auditing is essential because mislabeling or protocol variation in a small number of rare cases can disproportionately influence a federated model. Federated approaches should therefore include harmonized labels, quality control, privacy safeguards and site-level performance reporting.

Multimodal AI and foundation models may help connect radiology, histopathology, immunohistochemistry, genomics and outcomes. Yet rare subtype validation remains essential. A foundation model trained on large general pathology or common breast cancer datasets may not perform safely in rare breast cancer unless tested specifically in those populations.

Prospective validation should become a priority. Possible designs include registry-based validation, silent deployment, clinician-in-the-loop studies, tumor board impact studies and adaptive validation as rare cases accrue. Evaluation should address clinical usefulness, safety, workflow impact, patient-relevant outcomes and equity, not only discrimination metrics.

Patient-centered evaluation should become a core future direction. Rare breast cancer AI should be assessed not only by discrimination metrics but by whether it reduces diagnostic delay, improves access to expert review, supports clearer communication, improves trial matching, and respects patient preferences about AI-supported decision-making. Equity should also be evaluated because rare cancer datasets may underrepresent male patients, older adults, minority populations, and patients treated outside high-resource cancer centers.

Regulatory science should address the special case of small-cohort AI. Future policy should clarify how evidence thresholds, real-world monitoring, synthetic data documentation, model updates and post-market surveillance can be adapted for rare cancers without lowering patient-safety standards. This is especially important for tools that combine imaging, pathology, molecular testing and clinical decision support across jurisdictions.

### What should not be done at this time

A governance framework should define boundaries as clearly as opportunities. AI models for rare breast cancers should not be deployed as autonomous diagnostic or treatment-selection systems without prospective clinical oversight. Synthetic data should not be used as a substitute for real external validation, and synthetic cases should not be included in test sets used to claim clinical performance. Small retrospective datasets should not support broad claims of generalizability across rare subtypes, institutions, imaging protocols, ethnic groups or treatment settings.

Investigators should also avoid presenting pooled pan-rare performance as proof that every rare subtype is safe to use. Saliency maps or feature-importance plots should not be treated as evidence of biological validity unless their stability and clinical plausibility have been assessed. Large language models may assist summarization, evidence retrieval or tumor board preparation, but they should not generate unverified treatment recommendations or replace specialist judgment. These restrictions are not barriers to innovation; they are safeguards that keep the clinical claim aligned with the evidence.

## Conclusion

Rare breast cancers expose an important limitation of current oncology AI: many models are built under assumptions that rare diseases cannot easily satisfy. Large datasets, balanced classes, stable labels and independent validation cohorts are often unavailable. As a result, AI models in rare breast cancers face heightened risks of overfitting, unstable performance, biased outputs, misleading synthetic data effects and weak generalizability.

At the same time, rare breast cancers are precisely the type of clinical problem where responsibly designed AI may help. AI could support earlier recognition of atypical imaging patterns, assist histopathological review, integrate molecular and clinical data, improve risk stratification and strengthen multidisciplinary tumor board decision-making. These opportunities are especially relevant in precision oncology, where rare cancers often require individualized interpretation beyond standard treatment algorithms.

This manuscript proposes a five-domain governance framework for AI in rare breast cancers: intended clinical use, small-cohort validation, synthetic data governance, human oversight and lifecycle monitoring. Synthetic data may help address data scarcity, but it should not replace validation on real clinical cases. The central principle is that the strength of the AI claim must match the strength of the evidence.

For rare breast cancers, the future of AI should not be defined by model complexity alone. It should be defined by the ability to generate clinically trustworthy decision support under conditions of rarity, uncertainty and high patient impact. With these safeguards, AI can evolve from an aspirational technology into a clinically trustworthy partner in rare breast cancer precision oncology.

## Data Availability

No datasets were generated or analysed during the current study.
